# Computational Intelligence‐Assisted Understanding of Nature‐Inspired Superhydrophobic Behavior

**DOI:** 10.1002/advs.201700520

**Published:** 2017-12-08

**Authors:** Xia Zhang, Bei Ding, Ran Cheng, Sebastian C. Dixon, Yao Lu

**Affiliations:** ^1^ National & Local Joint Engineering Research Center for Applied Technology of Hybrid Nanomaterials Henan University Kaifeng 475004 P.R. China; ^2^ Department of Chemistry University College London 20 Gordon Street London WC1H 0AJ UK; ^3^ School of Computer Science University of Birmingham Birmingham B15 2TT UK; ^4^ Nanoengineered Systems Laboratory UCL Mechanical Engineering University College London London WC1E 7JE UK

**Keywords:** artificial neural networks, computational intelligence, evolutionary computation, superhydrophobic behavior

## Abstract

In recent years, state‐of‐the‐art computational modeling of physical and chemical systems has shown itself to be an invaluable resource in the prediction of the properties and behavior of functional materials. However, construction of a useful computational model for novel systems in both academic and industrial contexts often requires a great depth of physicochemical theory and/or a wealth of empirical data, and a shortage in the availability of either frustrates the modeling process. In this work, computational intelligence is instead used, including artificial neural networks and evolutionary computation, to enhance our understanding of nature‐inspired superhydrophobic behavior. The relationships between experimental parameters (water droplet volume, weight percentage of nanoparticles used in the synthesis of the polymer composite, and distance separating the superhydrophobic surface and the pendant water droplet in adhesive force measurements) and multiple objectives (water droplet contact angle, sliding angle, and adhesive force) are built and weighted. The obtained optimal parameters are consistent with the experimental observations. This new approach to materials modeling has great potential to be applied more generally to aid design, fabrication, and optimization for myriad functional materials.

## 1 Introduction

Superhydrophobic surfaces have attracted much attention from the scientific community and captured the imagination of the public due to their various useful applications including anti‐corrosion,^1^ anti‐icing,^2^ oil–water separation,^3^ and so on. Among these, self‐cleaning is one of the most significant applications.^4^ The lotus leaf is an excellent example of a superhydrophobic surface found in nature, upon which water droplets will exist in a spherical form rather than wetting the leaf by spreading out or staining the surface of the leaf; when such superhydrophobic surfaces are tilted slightly, sessile water droplets will roll off easily without leaving any trace of wetting, while the rolling motion of the droplet collects dirt from the surface, in such a way that lotus leaves can be described as “self‐cleaning.”^5^ To achieve superhydrophobicity, the lotus leaf surface has micrometer‐scale morphology combined with nanoscaled waxy fibrils to enable an extremely low affinity to water droplets.^6^ On such surfaces water droplets are able to bounce, and the contact time between bouncing water droplets and superhydrophobic surfaces becomes a key factor of their dynamic characterization.^7^ The water bouncing motion is well studied by physicists and the contact time can be further reduced by tuning the surface micromorphology and droplet Weber number to achieve “nonaxisymmetric bouncing,”^8^ “pancake bouncing,”^9^ and “sausage bouncing”^10^ of water droplets.

In the static characterization of superhydrophobic surfaces, the three most important measurements are water contact angle (CA), sliding angle (SA), and adhesive force.^11^ As shown in Figure S1 (Supporting Information), imagining a side‐on view of a superhydrophobic surface, a water droplet coming into contact with the solid surface creates an angle between the flat region of surface contact at the base of the droplet and a tangent drawn along the outer edge of the droplet at the point where liquid, solid, and air meet; this angle is widely identified as the water droplet contact angle and provides a quantifiable metric for the wettability of a solid substrate. Usually, a larger water contact angle correlates with a greater degree of superhydrophobicity.^12^ Meanwhile, the droplet sliding angle quantifies the mobility of a water droplet on a given surface, defined as the maximum angle at which a level surface can be tilted before a sessile water droplet will begin to slide.^13^ The sliding angle is also referred to as the rolling angle or tilting angle in some literature.^14^ Last, the water adhesive force defines the strength of interaction between a water droplet and a solid surface; it is measured at the instance when a water droplet separates from the surface, while the adhesive force is observed to vary with the distance that the droplet/substrate travels.^15^ To achieve self‐cleaning, the water contact angle is expected to be larger than 150° to minimize wetting, while the sliding angle and the adhesive force ought to be as small as possible so that water droplets can easily roll off the surface.^16^

The measurements of water contact angle, sliding angle, and adhesive force (the “objectives”) are generally considered standard characterization for a superhydrophobic surface. However, in practice the outcome of these measurements is subject to experimental variables such as the degree of nanostructure within the hierarchical surface morphology,^17^ water droplet volume, and the distance that the droplet or the surface travels when measuring the adhesive force (the “parameters”).^18^ For example, larger droplets are usually selected in sliding angle measurements for their ease of mobility, resulting in a smaller sliding angle; however, the details of the relationship between sliding angle and droplet size are not well understood, and it is unclear what effect a changing droplet size will have on the other objectives. Similar such uncertainties arise with the selection of parameters in the determination of the other objectives; increasing the degree of nanostructure reduces the contact angle, but what effect will this have on sliding angle or adhesive force? Complications arise in obtaining a conclusive characterization when all of said parameters and objectives are involved at the same time. It is observed that multiple experimental parameters tend to influence the determination of each objective, as such it is important to find out to what degree each parameter influences each objective, which parameter dominates in each instance and whether the experimental objectives conflict with each other. With limited experimental data and no analytical model available, approaching the problem using traditional mathematical tools presents difficulties.

Computational intelligence (CI), as a major branch of artificial intelligence, has been widely applied to solving complex real‐world problems^19^ and designing functional materials in fields such as energy science^20^ and catalysis.^21^ Computational techniques which are constructed using in‐depth physical and chemical theory are limited in their applicability to the wider design of other functional materials and processes where theory is cumbersome or less well‐understood, such as superhydrophobic surfaces. In such instances, the adoption of nature‐inspired CI where artificial neural networks and evolutionary algorithms enable location of optimal minima in multivariable systems has shown itself to be a promising alternative in solving global optimization problems.

In this work, SiO_2_ nanoparticles and polyvinyl chloride (PVC) were used to prepare biomimetic superhydrophobic surfaces with varying SiO_2_ nanoparticle content. Water contact angle, sliding angle, and adhesive forces were measured on these surfaces with varying droplet volumes. Adoption of CI related techniques was sought in order to investigate the relationship between experimental parameters, such as water droplet size, weight percentage of nanoparticles in the polymer composite and distance moved by the substrate in the measurement of droplet adhesive force, and experimental objectives, such as water contact angle, sliding angle, and the droplet adhesive force. In the computational process, artificial neural networks and evolutionary computation were applied in analyzing the experimental data. The artificial neural networks are inspired by the biological neural networks within the brains of animals and have been widely applied to learning and prediction tasks,^22^ while the evolutionary computation is inspired by natural evolution and has been widely applied to optimization tasks.^23^ First, a computational model is formulated to demonstrate the relationship between the experimental parameters and objectives. Second, artificial neural networks are adopted in building the surrogate models to estimate the functional mapping between the experimental parameters and objectives. Finally, evolutionary computation carries out multiobjective optimization on those surrogate models, leading to an optimal set of solutions to the experiments. Potential conflicts between the experimental objectives are likewise sought via the evolutionary multiobjective optimization. This method does not require large data sets and could potentially be applied more broadly to aid in the design, fabrication, and optimization of myriad functional materials.

## 2 Results and Discussion

**Figure 1:**
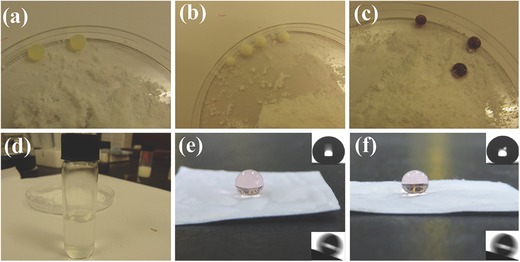
a) Juice, b) milk, and c) coffee droplets formed spheres on hydrophobic SiO_2_ powder. d) SiO_2_ powder dispersed in n‐hexane solution. e,f) Sessile water droplet on superhydrophobic SiO_2_/PVC surfaces: e) Sample S1; f) Sample S2, with inset contact angle (upper right) and sliding angle (lower right) measurements.

**Figure 2:**
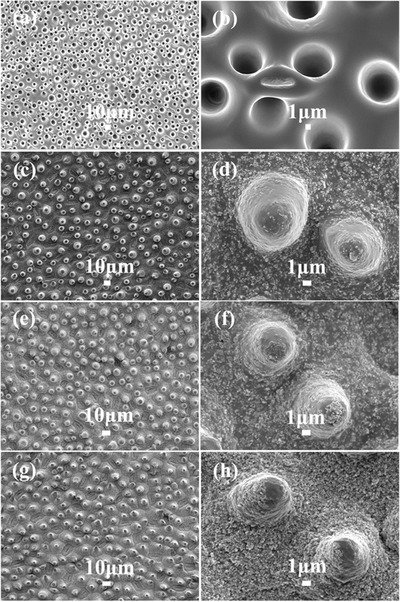
FESEM images of a,b) the obtained PDMS surface with negative biomimetic lotus topography and SiO_2_/PVC surface: c,d) Sample S1, e,f) Sample S3, and g,h) Sample S5.

Superhydrophobic samples were prepared by introducing hydrophobic silica (SiO_2_) nanoparticles into micrometer‐structured PVC. The hydrophobic SiO_2_ nanoparticles were obtained using a previously reported technique.^24^ The hydrophobic SiO_2_ nanoparticles showed high repellence to water and water‐based liquids, such as juice, milk, and coffee droplets as shown in **Figure** **1**a–c. The dispersibility of these SiO_2_ nanoparticles in organic media is illustrated in Figure 1d, in which the hydrophobic SiO_2_ nanoparticles form a stable dispersion in n‐hexane. The hydrophobic SiO_2_ nanoparticles were then mixed with PVC at five different ratios as shown in **Table** **1**, defining Samples S1–S5. The spherical form of water droplets on contact with Samples S1 and S2 is displayed in Figure 1e,f respectively. To create microscaled morphology, a lotus leaf was used as a template to transfer its micrometer structures onto the SiO_2_/PVC coatings as shown in Figure S2 (Supporting Information). Polydimethylsiloxane (PDMS) solution was poured onto a lotus leaf; after curing, the lotus leaf was removed leaving a negative topography on the PDMS template. The SiO_2_/PVC dispersion was poured onto the negative PDMS template; after the SiO_2_/PVC dispersion had dried, the PDMS was removed to yield the finished SiO_2_/PVC composite. **Figure** **2** shows the field emission scanning electron microscopy (FESEM) images of (a,b) the negative PDMS template and (c–h) the SiO_2_/PVC surfaces. It is evident that the microscopic structure of the lotus leaf was successfully transferred to the SiO_2_/PVC surfaces. Figure 2c–h compared the surface morphologies of Samples S1, S3, and S5; the micrometer structures on these samples are of the similar sizes as they all “copy” the same topographies from the lotus leaf. However, Samples S1, S3, and S5 show different ratios of nanoscaled structures due to the varying ratios of SiO_2_ nanoparticles added to the PVC, where Sample S5 had the highest degree of nanoscaled structure (SiO_2_: 46.7 wt%) while Sample S1 had the least (SiO_2_: 20 wt%).

**Table 1:** The ratio of hydrophobic silica nanoparticles and PVC, defining Samples S1–S5. SiO_2_ wt% = m(SiO_2_)/[m(SiO_2_) + m(PVC)] × 100%

Weight content	Sample 1 (S1)	Sample 2 (S2)	Sample 3 (S3)	Sample 4 (S4)	Sample 5 (S5)
PVC (/g)	1.2	1.1	1.0	0.9	0.8
SiO_2_ (/g)	0.3	0.4	0.5	0.6	0.7
SiO_2_ wt%	20%	26.7%	33.3%	40%	46.7%

X‐ray photoelectron spectroscopy (XPS) was employed to investigate the chemical composition of the samples as shown in Figure S3 (Supporting Information). Only Si, O, C, and Cl were detected indicating the purity of the obtained SiO_2_/PVC film. From Samples S1–S5, it is clear that the amount of Si increased with the increasing addition of SiO_2_ nanoparticles.

**Figure 3:**
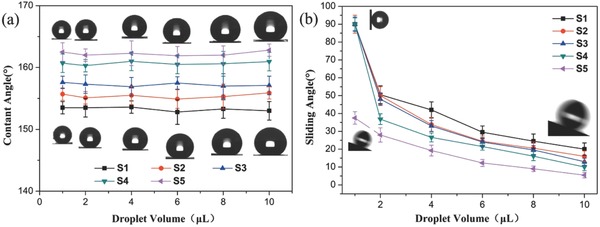
The relationship between water droplet volume and the a) contact angle and b) sliding angle for Samples S1–S5.

To characterize the wettability of Samples S1–S5, CA and SA were measured using water droplets at different volumes. **Figure** **3**a shows the plot of water contact angle as the droplet size increased for Samples S1–S5. The contact angle did not significantly change as the droplet size increased, while Sample S5 exhibited the highest contact angle correlating with the greatest addition of SiO_2_ nanoparticles among the five samples. Figure 3b illustrates the relationship between sliding angles and water droplet size. The sliding angle decreased as the increase of water droplet volume from 2 to 10 µL. Sample S5 achieved the lowest sliding angle among the five samples for all droplet volumes. Therefore, Sample S5 is considered to be the most superhydrophobic among the five samples under the evaluation of water contact angles and sliding angles. However, the influence of SiO_2_ nanoparticles and the droplet size on contact angle and sliding angle measurements has not been directly quantified.

**Figure 4:**
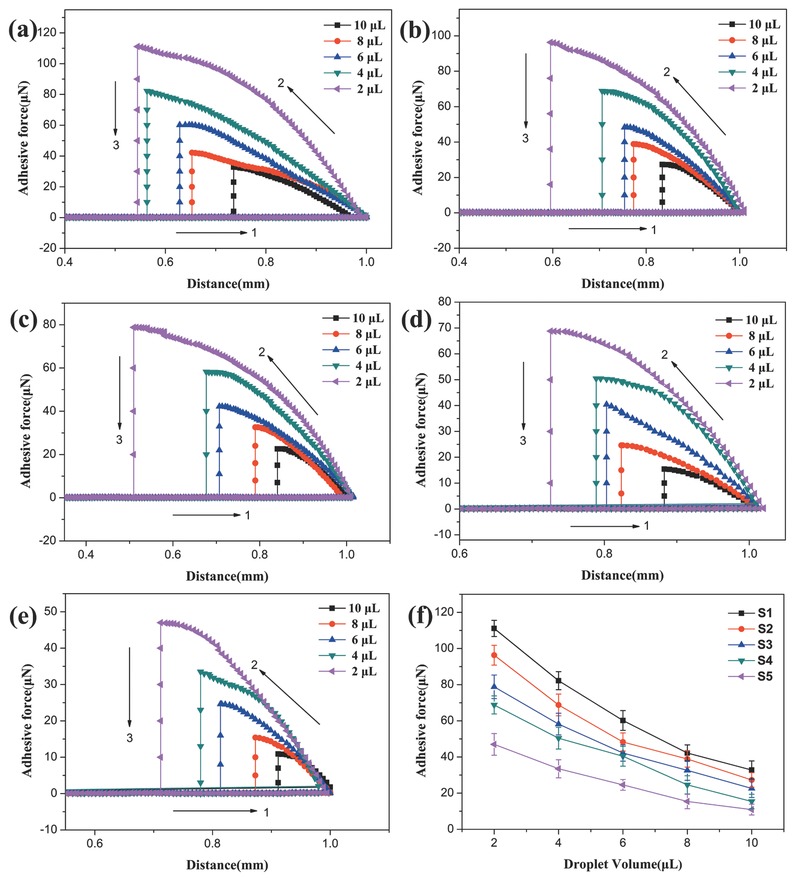
Adhesive force−distance curves with various water droplet volumes for Samples a) S1, b) S2, c) S3, d) S4, and e) S5. f) Adhesive force of water droplet on surface as a function of droplet volume.

To investigate the strength of interaction between water and the superhydrophobic coatings, a high‐sensitivity microelectromechanical balance system was used to measure the adhesive force between the surfaces of Samples S1–S5 and water droplets with different volumes. As shown in Figure S4 (Supporting Information), the water droplet was suspended by a copper ring, and the samples were positioned onto a stage under the water droplet. In the measurements, the stage moved up toward the suspended droplet at a rate of 0.03 mm s^−1^, then upon contact with the droplet, the stage moved downward at the same rate. Like so, the microelectromechanical balance collected data measuring the adhesive force of the droplet on the substrate. **Figure** **4** shows the adhesive force−distance curves for Samples S1–S5, respectively. Here, the distance refers to the travel distance of the stage. The force measurement process can be understood in three steps: in Step 1, there is zero adhesive force measured while the sample is moved upward toward the suspended droplet. The beginning of Step 2 is marked by the instance at which the droplet makes contact with the sample. During this step, samples on the stage were moved away from the droplet, enabling the collection of adhesive force data. Step 3 is marked by the separation of the sample from the droplet, at which point the measured adhesive force drops to 0. It can be seen that a smaller droplet volume tended to yield a greater adhesive force. Figure 4f shows the relationship between adhesive force and droplet size for Samples S1–S5, where Sample S5 exhibited the lowest adhesive force at each droplet volume. However, illustrating the multidimensional relationship between droplet volume, distance moved by the substrate in the adhesive force measurement, quantity of SiO_2_ nanoparticles incorporated into the polymer composite and the measured adhesive force is nontrivial, and optimization of this as well as other such multiparameter problems points toward assistance by CI techniques.

## 3 Computational Intelligence‐Assisted Analyses

Although it has been shown possible to plot “one to one” relationships between objectives such as water droplet contact angle, sliding angle, adhesive force, and experimental parameters, such as the nanoparticle‐to‐polymer weight ratio in the synthesis, water droplet size, and force measurement distance, it is clear that the current approach encounters difficulty with quantifying the influence of each factor. In addition, when attempting to gauge the simultaneous effect of two or more parameters such as nanoparticle ratio and droplet size, using the current approach it is impossible to determine a weighted contribution from each parameter on an objective such as droplet sliding angle. Here, computational intelligence‐assisted analysis was employed to understand the correlation between each experimental parameter and objective, based on data from the measurements carried out in Section 2. All computational analysis is performed in the Matlab software platform.

### 3.1 Problem Formulation

To achieve the best superhydrophobicity and self‐cleaning ability, the objectives to be optimized are defined in the computation such that the maximum contact angle, minimum sliding angle, and minimum adhesive force are obtained. The computational model is constructed as follows

(1) $$\{\begin{array}{c} \underset{x_{1},x_{2}}{\max}Y_{1}=f_{1}\left(X_{1},X_{2}\right)\\ \underset{x_{1},x_{2}}{\min}Y_{2}=f_{2}\left(X_{1},X_{2}\right)\\ \;\;\;\;\;\underset{x_{1},x_{2},x_{3}}{\min}Y_{3}=f_{3}\left(X_{1},X_{2},X_{3}\right) \end{array}$$

where, as summarized in **Table** **2**, *Y*_1_, *Y*_2_, and *Y*_3_, as the experimental objectives to be optimized, denote the contact angle, sliding angle, and adhesive force respectively, for which the functional mappings *f*_1_, *f*_2_, and *f*_3_ are constructed using the input variables *X*_1_, *X*_2_, and *X*_3_, which represent the experimental parameters: nanoparticle‐to‐polymer weight percentage, water droplet volume, and distance travelled in the adhesive force measurement, respectively.

**Table 2:** Symbols, denotations, and their scales in the problem formulation

Symbol	Meaning	Range
*X*_1_	Percentage of SiO_2_ in SiO_2_/PVC mixture	20–46.7 wt%
*X*_2_	Water droplet volume	2–10 µL
*X*_3_	Distance	0–1.0 mm
*Y*_1_	Contact angle	≤180°
*Y*_2_	Sliding angle	0–90°
*Y*_3_	Adhesive force	>0 µN

In order to quantify the correlations between the experimental parameters and objectives, namely *X*_1_, *X*_2_, *X*_3_, *Y*_1_, *Y*_2_, and *Y*_3_, statistical correlation analysis is performed on the experimental data obtained in Section 2. The correlation coefficient *r_xy_* between any experimental parameter *X* and any experimental objective (or parameter) *Y* is calculated as

(2) $$r_{xy}=\frac{\sum_{i=1}^{n}{\left(x_{i}-\bar{x}\right)}^{}\left(y_{i}-\bar{y}\right)}{\sqrt{\sum_{i=1}^{n}{\left(x_{i}-\bar{x}\right)}^{2}\sum_{i=1}^{n}{\left(y_{i}-\bar{y}\right)}^{2}}}$$

where *n* is the number of experimental data points obtained in Section 2 for *X* and *Y*, while $\bar{x}$ and $\bar{y}$ are the mean values of *X* and *Y*, respectively. As a result, the three correlation matrices for the functional mappings *f*_1_, *f*_2_, and *f*_3_ for the optimization of *Y*_1_, *Y*_2_, and *Y*_3_ respectively are obtained

(3) $$R_{1}=\;\left[\begin{array}{ccc} 1 & 0 & 0.99\\ 0 & 1 & 0.02\\ 0.99 & 0.02 & 1 \end{array}\right]$$

(4) $$R_{2}=\left[\begin{array}{ccc} 1 & 0 & -0.50\\ 0 & 1 & -0.83\\ -0.50 & -0.83 & 1 \end{array}\right]$$

(5) $$R_{3}=\left[\begin{array}{cccc} 1 & -0.05 & 0.27 & -0.30\\ -0.05 & 1 & 0.34 & -0.58\\ 0.27 & 0.34 & 1 & -0.88\\ -0.30 & -0.58 & -0.88 & 1 \end{array}\right]$$

where each element *r_ij_* in *R_k_* is either, when *i* ≤ *j* < *D* (with *D* denoting the size of the matrix), the correlation coefficient between experimental parameters *X_i_* and *X_j_*, or when *j* = *k*, between the experimental parameter *X_i_* and experimental objective *Y_k_*.

According to *R*_1_, the correlation coefficient for *X*_1_ and *X*_2_ is 0, which means that those two experimental parameters, namely the weight percentage of SiO_2_ in the polymer and the water droplet volume, are independent of each other, which means that adjusting one in an experiment should not affect measurements made in which the other is the measured variable. Another interesting observation is that nanoparticle quantity *X*_1_ is also strongly positively correlated with the water droplet contact angle *Y*_1_, as indicated by the correlation coefficient 0.99, while the water droplet volume *X*_2_ is virtually independent of its observed contact angle *Y*_1_, indicated by a correlation coefficient of 0.02.

Those experimental parameters *X*_1_ and *X*_2_ remain uncorrelated according to *R*_2_. By contrast, both *X*_1_ and *X*_2_ show negative correlations to the sliding angle *Y*_2_, indicated by the correlation coefficients of −0.50 and −0.83, respectively.

While *R*_1_ and *R*_2_ involve only two experimental parameters, *R*_3_, which involves *X*_1_, *X*_2_, and *X*_3_, is more complicated. As indicated by the correlation coefficients of −0.30, −0.58, and −0.88 in *R*_3_, all of *X*_1_, *X*_2_, and *X*_3_ are negatively correlated with the experimental objective *Y*_3_, namely, the adhesive force. This means that the measured adhesive force is changed by adjusting any of those three experimental parameters, while adjusting the distance moved has the most significant impact.

### 3.2 Artificial Neural Network‐Based Surrogate Modeling

In order to obtain surrogate models for the functional mappings *f*_1_, *f*_2_, and *f*_3_, artificial neural networks were trained by the experimental data obtained in Section 2. As shown in Figure S5 (Supporting Information), the neural networks adopted for building the surrogate models for *f*_1_, *f*_2_, and *f*_3_ are the classic two‐layer feed‐forward networks with sigmoid hidden neurons *S*_sigmold_ (***z***) and linear output neurons *S*_linear_ (*z*)^25^

(6) $$S_{\mathrm{sigmoid}}\left(z\right)=\frac{1}{1+e^{-\left(z\right)}}$$

(7) $$S_{\mathrm{linear}}\left(z\right)=z$$

where *z* = *wx* + *b* is the weight sum of the input *x*, and *w* and *b* are known as the weight matrix and bias vector, respectively.

**Figure 5:**
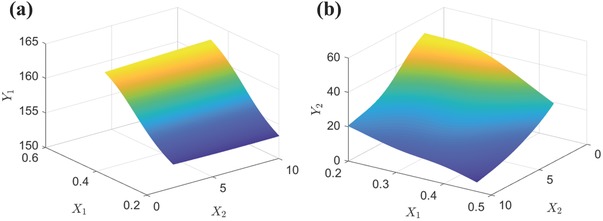
The functional mapping landscapes of a) *f*_1_ and b) *f*_2_ obtained by the surrogate models built by artificial neural networks. [*X*_1_, percentage of SiO_2_ in SiO_2_/PVC mixture (wt%); *X*_2,_ water droplet volume (*µ*L); *Y*_1_, water contact angle (°); *Y*_2_, sliding angle (°)].

In order to train the neural networks as per Figure S5 (Supporting Information), the experimental data obtained in Section 2 is used as training data, and the Bayesian regularization is used as the training function. As shown in Figure S6 (Supporting Information), all three neural networks returned small training errors, indicating a high degree of accuracy for the regressions. For further assessment, functional mapping landscapes of *f*_1_ and *f*_2_ using the surrogate models obtained by the neural networks have been plotted in **Figure** **5**. It can be observed that the correlations between *X*_1_, *X*_2_ and *f*_1_, *f*_2_ are consistent with the correlation analysis performed in Section 3.1. Using the surrogate models of *f*_1_, *f*_2_, and *f*_3_ obtained by the neural networks, numerical optimization is performed to obtain the optimal experimental parameter settings in the following subsection.

### 3.3 Evolutionary Computation‐Based Multiobjective Optimization

Since there are three objective functions *f*_1_, *f*_2_, and *f*_3_ in the model formulated in Section 3.1, it is a typical multiobjective optimization problem,^26^ where there are more than one (conflicting) objectives to be optimized simultaneously.

In order to obtain solutions to such a problem, the evolutionary multiobjective optimization technique is applied.^26^ The state‐of‐the‐art reference vector‐guided evolutionary algorithm (RVEA)^27^ is employed, which has previously been successfully applied to other real‐world applications such as the optimization of hybrid electric vehicle control.^28^

**Figure 6:**
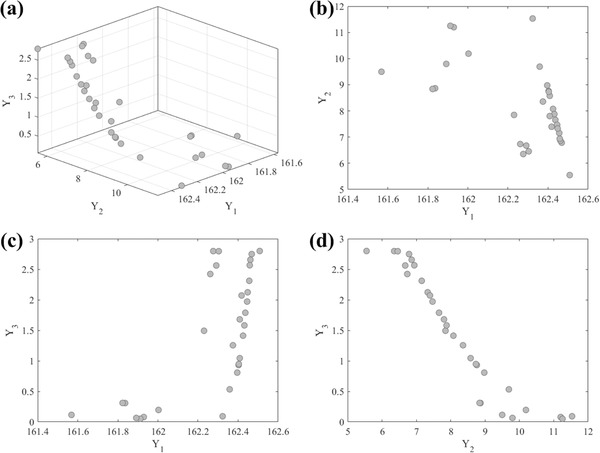
The final solution set obtained by optimising the surrogate models from Section 3.2 by using the state‐of‐the‐art RVEA. [*Y*_1_, water contact angle (°); *Y*_2_, sliding angle (°); *Y*_3_, adhesive force (*µ*N)].

As shown in **Figure** **6**a, RVEA has obtained a number of “nondominated” solutions by optimizing the surrogate models from Section 3.2. There are two important observations to make. First, as shown in Figure 6b,c, *f*_1_ can be always optimized regardless of the specific values of *f*_2_ and *f*_3_, resulting in an optimal value around *Y*_1_ = 162, which means that the contact angle is independent from sliding angle or adhesive force. Second, as shown in Figure 6d, *f*_2_ and *f*_3_ are conflicting with each other since an optimum cannot be reached simultaneously, thus resulting in a set of trade‐off solutions distributed in the range between *Y*_2_ ∈ (5, 12) and *Y*_3_ ∈ (0, 3), which means that the sliding angle and adhesive force are directly at odds and cannot coexist in a mutually optimal state. As such, the optimal experimental parameters obtained by RVEA are *X*_1_ ∈ (0.44, 0.47), *X*_2_ ∈ (8.2, 10), and *X*_3_ ≈ 1, which is consistent with the experimental results in Section 2. This yields a set of values which can be used to achieve the optimal synthesis and characterization of a superhydrophobic surface.

## 4 Conclusion

A lotus leaf was used as a template in patterning SiO_2_/PVC coatings in order to make biomimetic superhydrophobic surfaces. The degree of nanoscaled structure was varied by the weight percentage of SiO_2_ nanoparticles in the SiO_2_/PVC mixture. Water droplet contact angles, sliding angles, and adhesive forces were measured on these superhydrophobic surfaces using droplets of various known volumes. Computational intelligence techniques including artificial neural networks and evolutionary computation were applied to analyze the relationship between experimental parameters (weight percentage of SiO_2_ nanoparticles in the polymer, water droplet volume, and distance between the pendant droplet and the superhydrophobic surface in adhesive force measurements) and experimental objectives (water droplet contact angle, sliding angle, and adhesive force). Within the parameter ranges investigated, the following conclusions can be drawn:

[1] The percentage of hierarchical nanoscaled structure on the micrometer‐scaled surface pattern is positively correlated with the water droplet contact angle, with a correlation coefficient as high as 0.99. This conclusion is widely in agreement with the literature.^29^
[2] The water droplet volume has virtually no bearing on its contact angle, with a correlation coefficient of 0.02. This suggests that any error in the droplet volume dispensed during contact angle measurements for a superhydrophobic material should not significantly influence the outcome of the measurement.
[3] Both the percentage of nanoscaled structure incorporated into the polymer and the water droplet volume exhibit negative correlations with droplet sliding angles, with correlation coefficients of −0.50 and −0.83, respectively. Of the two parameters, water droplet volume has the greater impact on its sliding angle.
[4] The adhesive force of water droplets on the superhydrophobic materials is negatively correlated with all experimental parameters investigated, including the percentage of nanoscaled structure in the polymer, water droplet volume, and droplet‐surface separation distance in the force measurements, with correlation coefficients of −0.30, −0.58, and −0.88, respectively. The outcome of this observation is that the observed adhesive force can be varied by adjusting any of those three experimental parameters, while the distance used in the measurement has the most significant impact on its outcome.
[5] Water droplet contact angle is independent from its sliding angle or adhesive force.
[6] Water droplet sliding angle and adhesive force are conflicting properties and cannot be optimized simultaneously.
[7] To achieve the optimal synthesis and characterization of a superhydrophobic self‐cleaning surface with the maximum water droplet contact angle, minimum droplet sliding angle and minimum adhesive force between the surface and the droplet, the quantity of SiO_2_ nanoparticles used in the polymer composite should be between 44 and 47 wt%, droplet size should be between 8.2 and 10 µL and the separation distance used in the measurement of the adhesive force should be about 1.0 mm.

The use of artificial neural networks and evolutionary computation here does not require large sets of experimental data or any detailed physicochemical theoretical background in order to predict and optimize experiments, so that it can be generalized and broadly applied to tackle problems in other research fields and industrial processes. Computational networks and optimization can be further trained to enhance accuracy and expanded to encompass a wider numerical range for each variable and output (for example, increasing the droplet size investigated to 100 µL) when larger data sets are obtained and input into the system. This system has great potential to be generally applied to aid design, fabrication, and optimization for myriad functional materials.

## 5 Experimental Section

*Preparation of Hydrophobic Silica Powder*: Na_2_SiO_3_ was dissolved in DI water (50 mL) to form a 0.15 *m* solution (defined as Solution A). Then hydrochloric acid (25 mL, 0.18 *m*) was added into the stirred Solution A. After half of the hydrochloric acid had been added, hexamethyldisilazane (25 mL, 12.5 × 10^−3^ m) was added dropwise to Solution A together with the remaining hydrochloric acid. The resulting suspension was heated to 60 °C with stirring for 4 h. The suspension was separated into two phases upon cooling to room temperature, with white foam floating atop the liquid phase. The foam was purified by filtration and washed repeatedly using a solution containing DI water and ethanol until Cl*^−^* could not be detected by silver nitrate solution by visual examination. The filter cake was redispersed into a solution containing DI water and ethanol at a volume ratio of 1:1 to form an emulsion. The emulsion was then spray dried and hydrophobic SiO2 nanoparticles were obtained.

*Fabrication of Biomimetic SiO_2_/PVC Coatings*: Lotus leaf was used as a template to fabricate hierarchical SiO_2_/PVC surface comprising hierarchical micro‐ and nanoscaled structures, as shown in Figure S2 (Supporting Information). In order to first create a negative mould, PDMS precursors and cross‐linking agent were mixed (10:1 weight ratio) and degassed in a desiccator at room temperature for ≈3 h to release air bubbles from the mixture. Then, the PDMS mixture was poured onto the lotus leaf templates. After curing at 70 °C for 10 h, the PDMS was solidified and then peeled off from the template, forming a negative replica of the lotus leaf template. The PDMS negative mould was then used to create the microstructure for the SiO_2_/PVC composite, while the nanoscaled features were provided by the incorporated nanoparticles. In this process, SiO_2_ powder and PVC were dispersed in tetrahydrofuran (10 mL) then the mixed solution was poured onto the PDMS negative template. After drying at room temperature, the PDMS template was removed and thus the SiO_2_/PVC superhydrophobic coating was obtained.

*Characterization*: The surface morphology of the sample was characterized by FESEM (JSM‐6701F). XPS (Thermo Scientific ESCALAB 250Xi) was used to characterize the surface chemical compositions. Sessile water CA was acquired using a DSA‐100 optical contact‐angle meter (Kruss Company, Ltd, Germany) at room temperature (22 °C). The average CA value was determined by measuring the same sample at five different positions. SA was measured at five different positions on a sample to determine the average. The image of the water droplet on the surface was captured with a digital camera (NIKON, P600). The adhesive force was measured by a high‐sensitivity microelectromechanical balance (Data‐ physics DCAT 11, Germany). A water drop was first suspended with a metal cap which was fixed to the balance, and the substrate was placed on the balance stage. The stage was moved upward at a constant speed of 0.03 mm s^−1^, until the surface contacted the droplet. Then the stage was moved down. The water droplet was stretched from the spherical to the elliptical, and the force increased gradually up to maximum. When the surface broke away from the water droplet, the water droplet changed back to spherical and the force reduced down to zero quickly. The force when the droplet is just leaving, the surface is defined as the maximum adhesive force.

## Conflict of Interest

The authors declare no conflict of interest.

## Supporting information

Supplementary
