# Supplementary material for: Computational Intelligence‐Assisted Understanding of Nature‐Inspired Superhydrophobic Behavior
Source: Adv Sci (Weinh). 2017 Dec 8;5(1):1700520. doi: 10.1002/advs.201700520 (PMC5770681; doi:10.1002/advs.201700520)
Supplement: Supplementary file 1 — Supplementary [file ADVS-5-na-s001.pdf]

Copyright WILEY-VCH Verlag GmbH & Co. KGaA, 69469 Weinheim, Germany, 2017.

ADVANCED SCIENCE
Open Access

Supporting Information

for *Adv. Sci.,* DOI: 10.1002/advs.201700520

Computational Intelligence-Assisted Understanding of Nature-Inspired Superhydrophobic Behavior

*Xia Zhang, Bei Ding, Ran Cheng, Sebastian C. Dixon, and Yao Lu**

Copyright WILEY-VCH Verlag GmbH & Co. KGaA, 69469 Weinheim, Germany, 2016.

Supporting Information

Computational intelligence assisted understanding of nature-inspired superhydrophobic behavior

Xia Zhang, Bei Ding, Ran Cheng, Sebastian C. Dixon, and *Yao Lu**

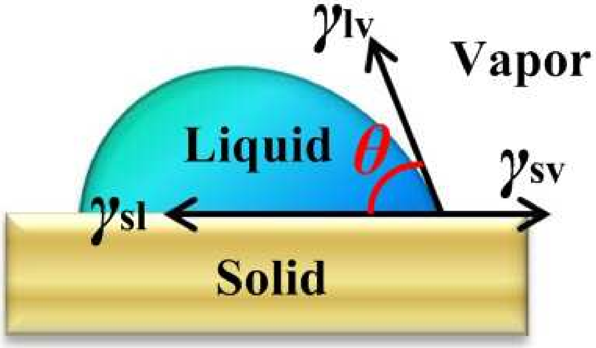

Figure S1. In Young's model, γ_{lv}, γ_{sv} and γ_{sl} refer to the interfacial tensions of liquid-vapor, solid-vapor, and solid-liquid phases, respectively. The contact angle θ is defined as $\cos\theta = (\gamma_{sv} - \gamma_{sl})/\gamma_{lv}$.

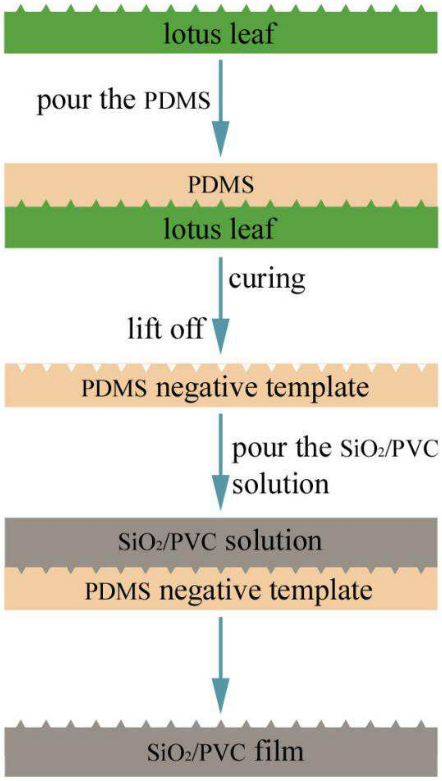

Figure S2. Schematic illustration of fabricating SiO_2/PVC film surface with positive lotus leaf surface topographies.

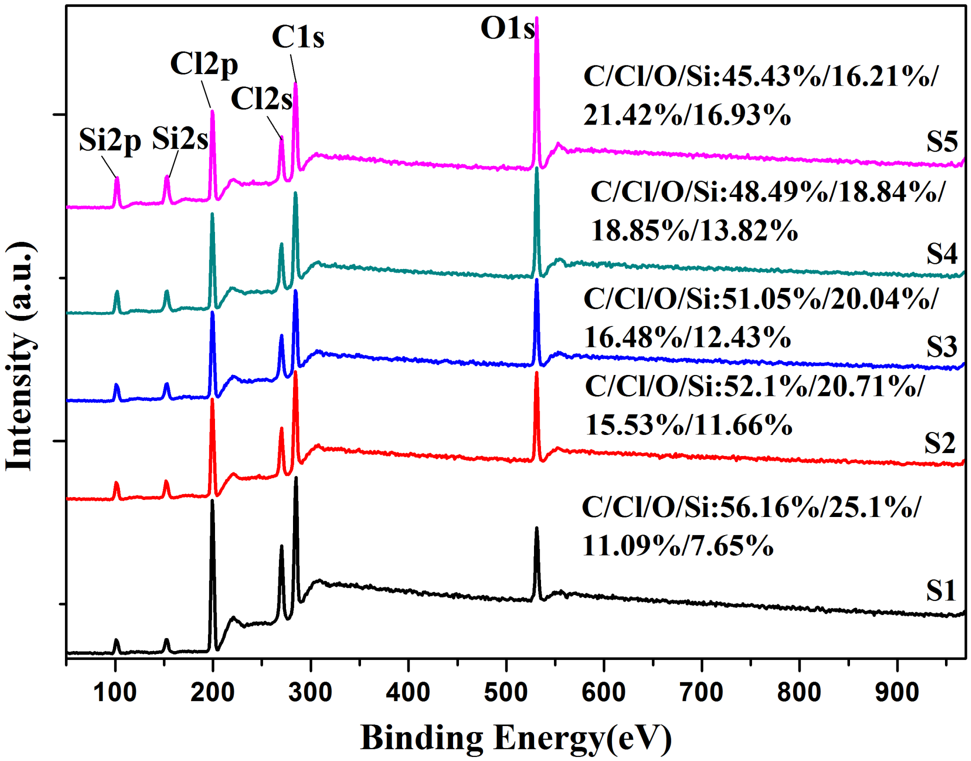

Figure S3. XPS survey spectra of Samples S1 to S5.

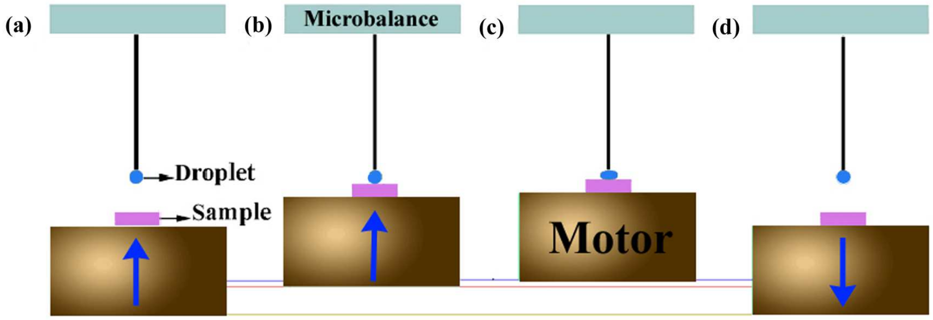

Figure S4. Schematic illustration of the micro-electromechanical balance collected data measuring the adhesive force of the droplet on the substrate. The water droplet was suspended by a copper ring and the samples were positioned onto a stage under the water droplet: (a) the stage (motor) moved up towards the suspended droplet at a rate of 0.03 mm/s (b and c) upon contact with the droplet and (d) the stage moved downwards at the same rate.

(a)
(b)

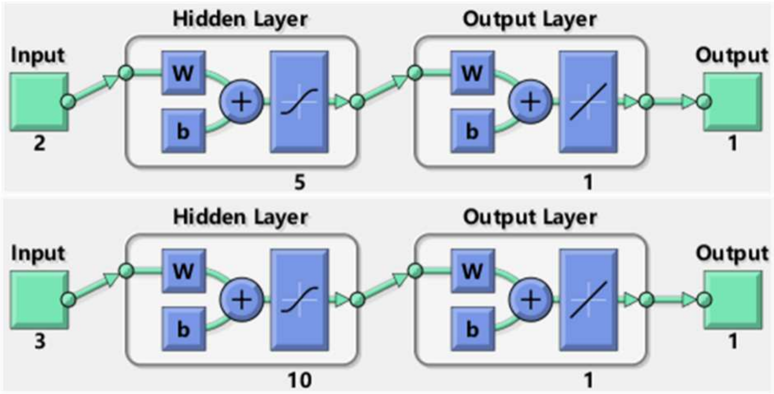

Figure S5. Structures of the networks used for modelling the functional mappings of f_1, f_2 and f_3. (a) Network structure for the regression of f_1 or f_2. Inputs are X_1 and X_2, and output is Y_1 or Y_2. The number of hidden neurons is 5. (b) Network structure for the regression of f_3. Inputs are X_1, X_2 and X_3, and output is Y_3. The number of hidden neurons is 10.

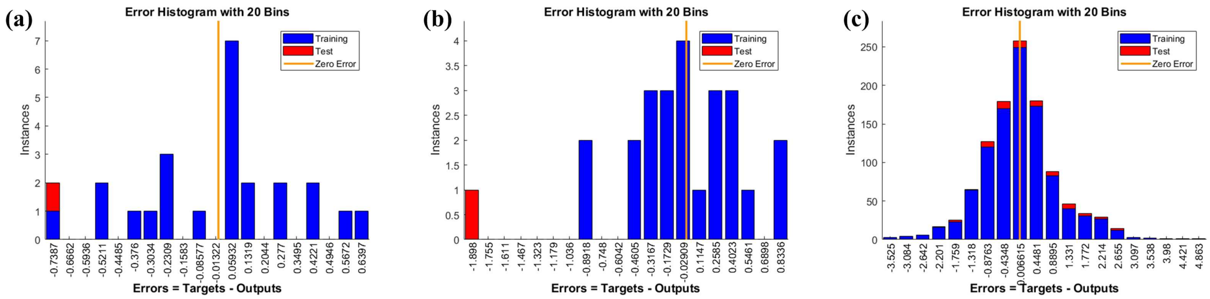

Figure S6. The training and test errors of the neural networks for the regressions of (a) f_1, (b) f_2 and (c) f_3, where the number of data instances in each dataset is 25, 25 and 1083, respectively. For each dataset 90%, 5% and 5% data instances are used as training, validation and test data, respectively.
